# Neural Correlates of Preference: A Transmodal Validation Study

**DOI:** 10.3389/fnhum.2019.00073

**Published:** 2019-03-18

**Authors:** Henrique T. Akiba, Marcelo F. Costa, July S. Gomes, Eduardo Oda, Paula B. Simurro, Alvaro M. Dias

**Affiliations:** ^1^Department of Psychiatry, Federal University of São Paulo, São Paulo, Brazil; ^2^Conselho Nacional de Desenvolvimneto Técnico e Científico—CNPq, Brasilia, Brazil; ^3^Fundação de Amparo à Pesquisa do Estado de São Paulo (FAPESP), São Paulo, Brazil

**Keywords:** liking, affective reactions, time-dependent measurement, EEG, ECG, eye tracking, neurocinematics

## Abstract

Liking is one of the most important psychological processes associated with the reward system, being involved in affective processing and pleasure/displeasure encoding. Currently, there is no consensus regarding the combination of physiological indicators which best predict liking, especially when applied to dynamic stimuli such as videos. There is a lack of a standard methodology to assess likeability over time and therefore in assessing narrative and semantic aspects of the stimulus. We developed a time-dependent method to evaluate the physiological correlates of likeability for three different thematic categories, namely: adventure (AV), comedy (CM), and nature landscape (LS). Twenty-eight healthy adults with ages ranging from 18 to 35 years (average: 23.85 years) were enrolled in the study. The participants were asked to provide likeability ratings for videos as they watched them, using a response box. Three 60-s videos were presented, one for each category, in randomized order while the participant’s physiological data [electroencephalogram (EEG), electrocardiogram (ECG) and eye tracking (ET)] was recorded. The comedy video (CM) presented the smallest minimum accumulated normalized rating (ANR; *p* = 0.013) and the LS video presented the highest maximum ANR (*p* = 0.039). The LS video presented the longest time for first response (*p* < 0.001) and the AV video presented the shortest time for maximum response (*p* = 0.016). The LS video had the highest mean likeability rating with 1.43 ± 2.31 points; and the CM video had the lowest with 0.57 ± 1.77. Multiple linear regression models were created to predict the likeability of each video using the following physiological indicators; AV: power in beta band at C4 and P4 (*p* = 0.004, adj. *R*^2^ = 0.301); CM: alpha power in Fp2 (*p* = 0.001, adj. *R*^2^ = 0.326) and LS: alpha power in P4, F8, and Fp2; beta power in C4 and P4 and pupil size, (*p* = 0.002, adj. *R*^2^ = 0.489). Despite its limitations (e.g., using one 1-min video per category) our findings suggest that there is a considerable difference in the psychophysiological correlates of stimuli with different contextual properties and that the use of time-dependent methods to assess videos should be considered as best practices.

## Introduction

The brain reward system is composed of three subsystems involving learning, emotional and motivational processing, according to Berridge and collaborators. Respectively, these components are responsible for reward learning, liking and wanting (Berridge, 2004; Berridge et al., 2009; Berridge and Kringelbach, 2013, 2015). All of them have explicit and implicit constituents and a vast number of studies have involved efforts to build bridges between the two, using purely neuroscientific or mixed strategies, which often involve fMRI (Silberstein and Nield, 2008; Kühn and Gallinat, 2012), electroencephalogram (EEG; Han et al., 2017), peripheral electrophysiology (Sánchez-Navarro et al., 2008) and eye tracking (ET; R.-Tavakoli et al., 2015).

Liking is recognized as one of the most important psychological processes associated with the reward system. It is associated with affective processing and pleasure/displeasure encoding, which provides feedback that guides the interaction with every stimulus which one encounters (Steiner et al., 2001; Berridge et al., 2009; Kringelbach and Berridge, 2010; Smith et al., 2011; Berridge and Kringelbach, 2013, 2015). The declarative component of liking can be measured through the explicit response of the subject; however, the non-declarative component requires a more specific approach, such as the psychophysiological evaluation of affective reactions or behavioral analysis.

Affective reactions can be described in terms of two fundamental dimensions: valence (pleasure/displeasure) and arousal (activation/inhibition; Russell, 1980, 2003; Gerber et al., 2008). These dimensions tend to relate to each other in “V” shape fashion; high arousal and positive valence encode high likeability, whereas high arousal associated with a negative valence leads to unlikeable experiences (Kuppens et al., 2013). Despite the large body of research on valence-arousal approach and its physiological correlates, the current literature is focused on a global assessment of the emotional phenomena, employing measures to rate the stimulus as a whole, such as the Self-Assessment Manikin (Bradley and Lang, 1994) or through visual analog scales.

While being appropriate for assessing static stimuli such as images, a global assessment may not be the best option to assess complex stimuli which change dynamically over time, such as videos. Audiovisual stimuli comprise the current mainstream form of media communication and perhaps the best resource for inducing affective reactions in a controlled and easily reproducible environment. Videos have better ecological validity than images and sounds, as they can embrace more comprehensive narratives over time, and are therefore closer to a real-life experience.

Time-dependent measures have been used to evaluate dynamic changes of perception in sensory evaluation for more than 80 years, starting with studies by Holway and Hurvich (1937) on taste perception. Time-dependent methods are a set of descriptive analysis techniques that allow the monitoring of changes in the temporal sensory profile of a stimulus. As a descriptive technique, it usually provides detailed, precise, reliable and objective information regarding the stimulus sensorial attributes. Unlike non-time dependent measures, which provide a global sensory profile of the stimulus, time-dependent measures are focused on capturing dynamic changes in one or more attributes, providing insightful data when detailed time information is needed (Hort et al., 2017). These methods have been consistently applied to assess affective responses such as liking for certain foods in studies since the 1990s (Taylor and Pangborn, 1990; Sudre et al., 2012; Jager et al., 2014; Thomas et al., 2015).

Surprisingly, very few authors have employed time-dependent measures, despite them being well established, to assess affective response induced by videos. In a recent systematic review conducted by our team on affective psychophysiological responses to videos stimuli (submitted article), we noted that only one study (Golland et al., 2014) employed time-dependent measures to assess the declarative components of affective reaction whilst investigating their psychophysiological correlates [i.e., EEG, electrocardiogram (ECG) and ET]. This is worth noting, as there is a known methodological limitation in evaluating the affective impact of video narratives in affective reactions by requiring subjects to recall and the experience they had throughout their duration, due to the so-called Primacy-Recency Effect in short-term memory (Henson, 1998), which is the tendency to remember information presented at the beginning and at the end of a series of events, hence overestimating these moments when declaring the likeability of a video.

Our review shows that most studies tend to look for general psychophysiological patterns, capable of distinguishing pleasant and unpleasant stimuli, with little relation to the contextual aspects of the stimuli. For example, it appears as if certain brainwaves and patterns of activation of the autonomous system have intrinsic value to the subject, as if they translated the occurrence of specific computations in the domain of pleasantness. In contrast to this, two very different experiences, such as watching a horror movie and taking part in a meditation session, may be rated and experienced as equally pleasant, in which the physiological correlates of each experience are expected to be extremely different, aligned with the phenomenological states that trigger the reaction. Feeling relaxed in a horror movie should indicate ineffectiveness, not the opposite, as a generalist approach to these correlates would in fact imply.

Considering that most physiological indicators of affective reactions induced by videos are established using non-time-dependent assessment methods as reference and that affect detection models tend to be more accurate while employing multimodal physiological evaluations (D’Mello and Kory, 2015), the most appropriate combination of physiological indicators to describe the neurobiological correlates of subjective liking using time-dependent measurements are still to be determined or at least further understood.

In light of those issues, the present article, a proof-of-concept study employing a time-dependent method to evaluate its associations to physiological measures, namely EEG, ECG and ET, illustrates how these indicators can be used to predict likeability in three categories of videos: adventure, comedy and observational (videos of nature landscapes).

## Materials and Methods

### Participants

This study involved healthy adults, aged 20–35 years old, with no history of neurological, psychiatric or cardiological diseases. To diminish socioeconomic biases, it was established that all participants were from socioeconomic classes A and B (middle class and above).

The following were included as exclusion criteria: individuals with scores higher than 7 on the SRQ-20, a self-evaluation psychiatric screening questionnaire (Mari and Williams, 1986), morbid obesity, strabismus, having slept fewer than 5 h the night before, chronic use of psychoactive drugs, or having used illegal drugs at least 3 days before the experiment. Thirty-five healthy adults were contacted for the first assessment, and 28 (18 males, mean age 23.85 years) were included on the study. Two participants had slept fewer than 5 h the night before the experiment and three individuals scored more than 7 points on the SRQ-20 questionnaire. Participants were recruited from the University of São Paulo (USP), through announcements made after classes at the university.

### Materials

#### Stimuli Selection and Presentation

Three movies, one from each thematic category, adventure (AV), comedy (CM) and nature landscape (LS), were selected from the Internet, following these selection criteria: (a) creative commons license or any license that allowed editing and reproducing for non-commercial purposes; (b) absence of verbal content; (c) at least 60 s of duration; and (d) 720p quality or higher. The selected videos were “Goliath Roller Coaster 4K POV Walibi Holland” (AV), “Centraal Beheer TVC 70–Acupuncture” (CM) and “Inukshuks under the stars” (LS).

Videos were edited so that they were all the same size and duration^1^. All videos were presented once to each participant in a pseudo-random order. Participants sat 60 cm from the screen and the stimuli were presented on a 15.1-inch LCD display with 1366 × 768 pixel resolution and 60 Hz refresh rate. The stimulus presentation software utilized was Tobii Studio 3.2.

#### Physiological Measures

Eye movements and pupillometry were recorded using Tobii X2–60 fixed ET, a binocular video-based eye tracker with 60 Hz sample rate. EEG and ECG recordings were made using g.tec gUSBAmp 3.0 from g.tec medical engineering, an FDA and CE certificated amplifier with 24 bits resolution and 16 channels. Electrophysiological data was collected at a 256 Hz sample rate. Twelve channels were used for EEG recordings and two channels for ECG recordings. EEG was recorded with g. SAHARA active system of dry electrodes and ECG was recorded using AgCl passive gel electrodes. EEG electrode positioning was based on the study by Yılmaz et al. (2014) and followed the international 10–20 system: Fp1, Fp2, F1, F2, F7, F8, C3, C4, P3, P4, O1, O2, with the reference positioned on the left mastoid and ground on the right mastoid. ECG followed bipolar positioning with the first active electrode above the right pectoralis major muscle, near the middle of the clavicle and the second electrode was positioned on the left superior portion of the abdomen, near the 7th rib. Reference was positioned symmetrically to the first active electrode, on the left chest and ground was positioned over the trapezius muscle. The limit of the impedance level for data collection was 5 kOhms or less.

#### Likeability Assessment

Likeability rating was assessed through an Arduino-based response box, with a rotating potentiometer attached to an encoder. Participants were instructed to rotate the potentiometer clockwise to increase the likeability rating or counterclockwise to decrease it, according to their experiences during the video. This device has small ticks, which provide tactile feedback to the participant while rotating it. The reason for using a potentiometer instead of a more conventional approach, like a slider or some rating button models, is that this piece can be rotated infinitely, which allows participants to rate without being constrained by upper or lower limits. A light sensor connected to the monitor was used to synchronize the stimulus presentation, physiological, and likeability data collection.

### Procedures

Experiments were conducted in a quiet, dimly lit room. Demographic data, socioeconomic data, education level, laterality, gaze dominance, hours of sleep, use of psychoactive drugs and history of psychiatric, neurological or cardiac diseases were all collected upon arrival. The SRQ-20 questionnaire was also filled out at this moment. Subsequently, participants were asked to sit in front of the monitor and the sensors were attached. After calibration and signal quality assessment, the baseline was recorded and the experiment was set to begin.

An instruction screen was presented telling the participant to rotate the potentiometer to indicate his liking of the videos as he watched them. He was told to the potentiometer clockwise to indicate that he liked the video and counterclockwise to indicate the opposite. In this sense, the more the participants rotated the potentiometer towards a direction, the more intense the corresponding experience (liking or disliking). Participants were also instructed to avoid moving or blinking excessively during the video presentations.

After being certain that each participant understood the instructions, the researcher left the room and the experiment started; a wireless doorbell was provided to call the researcher after the experiment was finished or in case of an issue. A 15-s resting interval was added between each video, to allow the participant to recover emotional homeostasis, avoiding carryover effects from one video to the next.

### Data Processing—Subjective Liking

Subjective liking was processed in three complementary analyses: accumulated normalized rating (ANR), sample percentage of response and moving rating sum. The purpose of the ANR was to highlight likeability trends over time. It was calculated by accumulating given ratings over time (for example, if the participant gave a +1 rating, by turning the potentiometer clockwise, in the second and third samples, and a −1 rating, by turning the potentiometer counterclockwise, in the fourth sample, the rating in the first five samples would be accumulated as follows: 0, 1, 1, 2, 1, 1). This procedure was done in the whole interval and z-scores for all samples for each individual were then calculated; this approach is especially sensitive to the narrative aspects of the video, capturing accumulated variability over time.

The purpose of the sample percentage of response was to create a manipulation test for the method, by assessing whether the participants presented similar response patterns to the stimuli over time, therefore evaluating its performance to assess group trends and providing validity measures for the method. It was calculated by dividing the data into 50% overlapped epochs of 1,000 ms (steps of 500 ms, therefore overlapping 50% of the previous time window, making a total of 90 epochs) and determining the percentage of the sample which presented responses in each epoch, regardless of valence (for example, the sample size was 28, so if 14 participants responded at least one time, during 10 s to 11 s interval, the sample percentage of response for this epoch was 50%. This procedure was repeated for all epochs).

**Figure 1:**
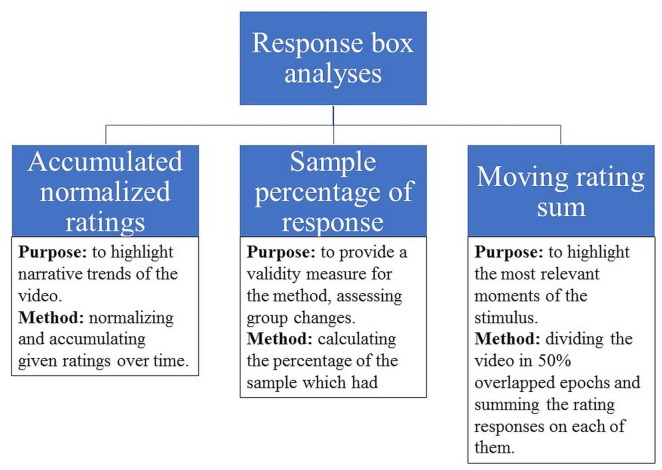
Likeability indicators processing summary.

The purpose of the moving rating sum was to assess granular likeability changes over time, highlighting the moments when the stimulus was most relevant. It was calculated by totaling the number of responses in 50% overlapped epochs of 500 ms, comprising 238 epochs. Each epoch was considered independently and each response that indicated liking (rotating clockwise) was coded as +1 and each response that indicated disliking (rotating counterclockwise) was coded as −1 (for example, if a participant rotated the potentiometer four times in 500 ms, rating +1, +1, +1 and −1, the final rating for this epoch would be +2. This procedure was repeated for all epochs). By these means, it was possible to determine the moments of maximum and minimum likeability of each video (i.e., the most and the least liked moments of the video), which were used for subsequent physiological pattern comparison and the regression analysis. The purpose and processing methods for each indicator is summarized in Figure 1.

### Data Processing—Physiological Measures

Electrophysiological signals were filtered offline using finite impulse response bandpass filters (EEG 2–40 Hz and ECG 2–100 Hz) and a notch filter (60 Hz). Blinks and other eye movements were removed using independent component analysis and noisy channels were interpolated using spherical interpolation. Finally, the signal was visually inspected and sections with artifacts were removed. EEG data preprocessing was made using EEGLAB 14.1 (Delorme and Makeig, 2004).

After data preprocessing, the spectral power of each EEG channel in each sample of the 1.5 s preceding the moments of maximum and minimum liking rating was calculated using wavelets transformation. The established frequency bands were: delta (2–4 Hz), theta (4–8 Hz), alpha (8–15 Hz), beta (15–30 Hz) and gamma (30–40 Hz). In order to calculate the mean power in each frequency band, the frequency vector was divided into 0.5 Hz bins and the average values within the frequency intervals were calculated; these procedures were made using Fieldtrip (Oostenveld et al., 2011). The selection of EEG variables to be included in the analysis was done using data of an aforementioned systematic review conducted by our group (submitted) on the psychophysiological correlates of affective reactions induced by videos; namely theta in the left frontal (Fp1, F1 and F7), right parietal (P4) and occipital regions (O1 and O2), alpha in the right frontal (Fp2, F2 and F8) and right parietal (P4), beta in the right frontal (Fp2, F2 and F8), right central (C4), parietal (P3 and P4) and left occipital region (O1) and gamma in the right frontal region (Fp2, F2 and F8).

Phasic cardiac response (PCR) in the 5 s preceding the moments of maximum and minimum liking ratings were calculated using Kardia, a Matlab-based toolbox for cardiologic data processing and analysis (Perakakis et al., 2010). To determine the PCR, the cardiac frequency was calculated on epochs of 200 ms and the data points were interpolated using cubic spline, as suggested by Guimarães and Santos (1998).

Pupil size was determined with Tobi Studio 3.2; this software automatically corrects pupil size according to the distance from the screen. Blinks and missing data were interpolated using Matlab 2015 cubic spline function. The mean pupil size in the 2 s preceding the moments of maximum and minimum liking rating was calculated and used for further analysis, as shown below.

### Statistical Analysis

**Figure 2:**
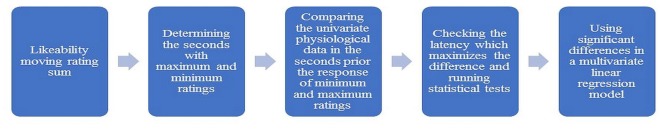
Physiological data processing and analysis.

The main objective of this exploratory, proof-of-concept, correlational study was to illustrate the use of a time-dependent approach to assess subjective liking and its physiological correlates to create predictive models. In this sense, the focus of the analysis of subjective liking measures is to highlight the differences between videos and the focus of the physiological univariate analysis was investigating the differences between the moments preceding the maximum and minimum likeability ratings (Supplementary Material), which were used in the regression analysis, as described in Figure 2. These comparisons were made using Friedman tests with *post hoc* Wilcoxon tests. In addition, these tests were performed for every epoch in order to provide a control measure and descriptive statistics were provided.

A multivariate linear regression model was created for each video, to predict the difference between the maximum and minimum likeability ratings, using as predictors the maximum differences on each physiological indicator in the moments prior to these epochs. Only indicators whose maximum differences were statistically significant at the univariate analysis (displayed in the Supplementary Material) were included in the regression analysis. In this sense, the AV video model included cardiac frequency and power in the following sites and frequency bands: theta in Fp1, F1 and O2; alpha in Fp2 and F2; beta in Fp2, F8, F2, C4, P3, P4, O1; and gamma in Fp2, F8, and F2. The CM video model included cardiac frequency, pupil size and power in the following sites and frequency bands: theta in Fp1, F7, F1 and O2; alpha in F1, Fp2, F8 and F2; beta in Fp2, F2, C4, P3, P4, O1; and gamma in Fp2, F8 and F2. The LS video model included, pupil size and power in the following sites and frequency bands: theta in F7, F1, P4 and O2; alpha in P4, Fp2 and F8; beta in Fp2, F8, F2, C4, P3, P4, O1; and gamma in Fp2, F8 and F2. All variables were normalized prior to the model creation and, due to a large number of variables and the sample size, we used a backward stepwise regression method.

The significance level for all tests was established at 95%. Friedman and Wilcoxon’s tests were conducted with Matlab Statistics and Machine Learning Toolbox and the Statistics Package for Social Sciences 21 was used for the linear regression model.

## Results

### Subjective Liking

#### Accumulated Normalized Ratings

Significant differences were observed in minimum (*p* = 0.013) and maximum ratings (*p* = 0.049). *Post hoc* tests showed that CM video minimum rating is significantly lower than AV video minimum rating (*p* = 0.04) while no significant differences were found in the pairwise comparison of maximum rating. In addition, significant differences were found between the time to first response in each video (*p* < 0.001) and the time of maximum rating (*p* = 0.016). *Post hoc* tests showed that the AV and LS videos’ times to first response are significantly lower than CM video time to first response (*p* < 0.001 and *p* = 0.023 respectively); and the time of maximum rating of the LS video is significantly higher than the AV video’s time (*p* = 0.027). No significant differences were found between mean rating, time for minimum response, number of responses and the difference between the minimum and maximum ratings. Results are presented in further detail in Table 1.

**Table 1:** Results of Friedman test with *post hoc* comparing subjective liking measurements.

	Mean AV	Mean CM	Mean LS	$\chi_{\left(2\right)}^{2}$	Sig.	AV-CM Sig.	AV-LS Sig.	CM-LS Sig.
Mean rating	0.38 ± 0.27	−0.06 ± 0.18	0.51 ± 0.27	0.271	0.873	−	−	−
Min. rating	−1.46 ± 1.71	−2.75 ± 3.2	−1.36 ± 1.61	8.614	0.013	0.04	1	0.158
Max. rating	3.71 ± 3.6	2 ± 1.96	4.43 ± 4.98	6.506	0.039	0.285	0.098	1
Min. time (s)	21.09 ± 18.5	21.57 ± 17	21.63 ± 18.7	0.13	0.937	−	−	−
Max. time (s)	13.27 ± 10.2	26.05 ± 19	22.82 ± 14.5	8.243	0.016	0.082	0.027	1
Time to first response	4.178 s	13.646 s	5.078 s	20.857	<0.001	<0.001	0.164	0.023
Number of responses	19.5	18.5	22	4.321	0.115	−	−	−
Max–min rating difference	5.18 ± 3.98	4.75 ± 3.98	5.79 ± 4.6	0.271	0.873	−	−	−

**Figure 3:**
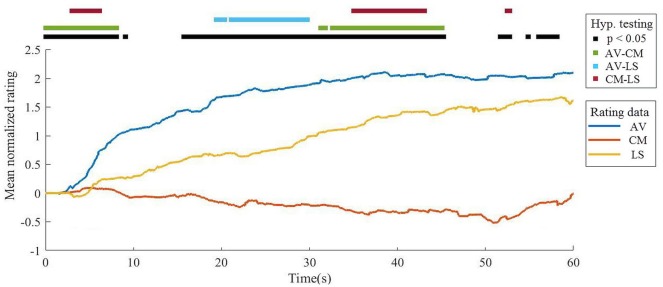
Comparison of mean accumulated normalized ratings (ANRs) by video. AV, adventure video; CM, comedy video; LS, landscape video.

Significant differences in liking were found among each video for nearly 65% of the video’s total duration, more specifically at the following intervals: 0–8.129 s; 9.023 s to 9.172 s; 51.66 s to 52.77 s; 54.85 s to 54.91 s and 56.04 s to 58.17 s, as presented in Figure 3. *Post hoc* tests have shown significant differences between all pairs in at least some moment in time. More specifically, AV-CM videos: at 0–8.129 s, 31.27 s to 31.87 s, 32.61 s to 43.91 s and 44.3 s to 45.13 s, approximately 34.62% of the stimuli duration, with a mean difference of 1.45 ± 0.889 points; AV-LS videos: at 19.4 s to 20.4 s and 21.2 s to 29.74 s, approximately 15.96% of the stimuli duration, with a mean difference of 0.702 ± 0.371 points; and CM-LS videos: at 3.008 s to 6.121 s; 35.01 s to 43.11 s and 52.47 s to 52.77 s, approximately 18.91% of the stimuli duration, with a mean difference of −0.680 ± 0.571 points. No significant differences were found between the global median of each video ($\chi_{\left(2\right)}^{2}$ = 4.357, *p* = 0.113).

#### Sample Percentage of Response

**Figure 4:**
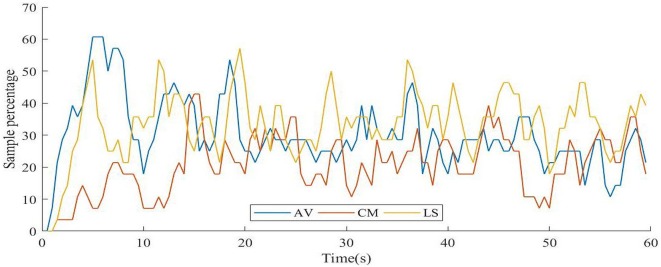
Sample percentage of response on each second. AV, adventure video; CM, comedy video; LS, landscape video.

AV video presented a mean sample percentage of the response of 30% ± 11% on each second, with a maximum of 61% between 5.5 s and 6.5 s, which is the moment just after the scene in which a rollercoaster goes downhill for the first time. The CM video presented a mean sample percentage of the response of 20% ± 9% in each second, with a maximum of 43% between 15.5 s and 16.5 s, which occurs during a scene in which acupuncture needles are being placed on the character. Regarding the LS video, the mean sample percentage was 34% ± 10% in each second, with a maximum of 57% between 19.5 s and 20.5 s, when a scene appears that features a beautiful landscape of tundra, river, and mountains on the horizon. The results for each video are presented in Figure 4.

#### Moving Rating Sum

**Figure 5:**
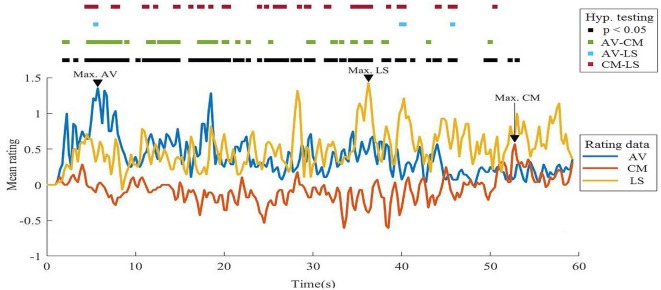
Comparison of mean likeability ratings on overlapped 500 ms epochs. AV, adventure video; CM, comedy video; LS, landscape video.

The maximum mean rating for the AV video was 1.36 ± 2.5 points at 5.75 s, corresponding to the same scene with the highest sample percentage of response; in the case of the CM video, the maximum mean rating was 0.57 ± 1.77 points at 52.75 s, which corresponds to the moment when the character has to decide whether to jump onto a safety net while his body is covered with needles or stay in a building on fire. Finally, in the LS video, the maximum mean rating was 1.43 ± 2.31 points at 36.25 s, which corresponds to a scene showing a stunning landscape with colorful flowers, mountains, and a lake. Significant differences were found in 47% of the duration of the stimulus. *Post hoc* tests showed differences between AV-CM, in 23%, AV-LS in 2% and CM-LS in 21% of the stimuli duration. The mean pair difference was 0.591 ± 0.339 points in AV-CM, 0.587 ± 0.354 points in AV-LS and −0.331 ± 0.390 points in CM-LS. The results for each video are presented in Figure 5.

### Regression Models

#### Adventure

A multiple linear regression was run to predict the likeability of the AV video based on EEG and ECG data. The multiple regression model significantly predicted the likeability rating for adventure, *F*_(2,25)_ = 6.822, *p* = 0.004, adj. *R*^2^ = 0.301, RMSE = 1.95. Two variables added statistically significant results to the prediction *p* < 0.05: beta power in C4 and P4. Regression coefficients and standard errors can be found in Table 2.

**Table 2:** Summary of multiple regression analysis of the likeability induced by the adventure video.

	Coef.		Std Coef.			Beta C.I. (95%)	
	Beta	Standard error	Beta	*t*	*p*-value	Lower threshold	Upper threshold
Intercept	3.917	0.716		5.471	#x0003C;0.001	2.443	5.392
C4 beta	2 × 10^−4^	69 × 10^−6^	0.468	2.903	0.008	58 × 10^−6^	34 × 10^−5^
P4 beta	19 × 10^−5^	8 × 10^−5^	0.392	2.431	0.023	3 × 10^−5^	36 × 10^−5^

*Coef: coefficients. Std: standardized*.

#### Comedy

A multiple regression was run to predict the likeability of the CM video from EEG, ECG and ET data. The multiple regression model statistically significantly predicted the likeability rating on comedy, *F*_(1,26)_ = 14,079, *p* = 0.001, adj. *R*^2^ = 0.326, RMSE = 1.91. Only one variable added significant results to the prediction *p* < 0.05: alpha power in Fp2. Regression coefficients and standard errors can be found in Table 3.

**Table 3:** Summary of multiple regression analysis of the likeability induced by the comedy video.

	Coef.		Std Coef.			Beta C.I. (95%)	
	Beta	Standard error	Beta	*t*	*p*-value	Lower threshold	Upper threshold
Intercept	3.794	0.668		5.685	<0.001	6 × 10^−6^	2.422
Fp2 alpha	−9 × 10^−5^	25 × 10^−6^	−0.593	−3.752	0.001	−14 × 10^−5^	−4 × 10^−5^

*Coef: coefficients. Std: standardized*.

#### Landscape

A multiple regression was run to predict the likeability of the landscape video from EEG, ECG, and ET data. The multiple regression model significantly predicted the likeability rating for the LS video, *F*_(6.21)_ = 5.304, *p* = 0.002, adj. *R*^2^ = 0.489, RMSE = 1.91. Six variables added significant results to the prediction *p* < 0.05: alpha power in P4, F8, and Fp2; beta power in C4 and P4 and pupil size. Regression coefficients and standard errors can be found in Table 4.

**Table 4:** Summary of multiple regression analysis of the likeability induced by the landscape video.

	Coef.		Std Coef.			Beta C.I. (95%)	
	Beta	Standard error	Beta	*t*	*p*-value	Lower threshold	Upper threshold
Intercept	4.209	0.875		4.808	<0.001	2.388	6.029
P4 alpha	5 × 10^−5^	69 × 10^−6^	0.502	3.216	0.004	2 × 10^−5^	9 × 10^−5^
F8 alpha	−2 × 10^−5^	8 × 10^−5^	−1.287	−4.373	<0.001	−4 × 10^−5^	−13 × 10^−6^
Fp2 alpha	−8 × 10^−5^	69 × 10^−6^	−0.448	−2.632	0.016	−15 × 10^−5^	−2 × 10^−5^
C4 beta	3 × 10^−4^	8 × 10^−5^	0.569	2.53	0.019	5 × 10^−5^	10 × 10^−5^
P4 beta	−25 × 10^−5^	69 × 10^−6^	−0.442	−3.004	0.007	−4 × 10^−4^	−34 × 10^−5^
Pupil size	−4.022	1.803	−0.541	−2.23	0.037	−7.773	−0.272

*Coef: coefficients. Std: standardized*.

## Discussion

The study of the affective reactions induced by complex stimuli such as videos and the dynamics of its psychophysiological correlates over time can be a challenging, yet important, task. Videos are among the most engaging and expensive media products and the use of time-dependent methods to assess affective reaction fluctuation scans provide valuable information which is not observable through conventional methods. This study is among the first to employ this kind of technique, providing a proof-of-concept on how to assess likeability and its psychophysiological correlates in three stimuli with marked contextual differences.

### Subjective Liking Assessment

The results of the first phase have shown that there are distinct patterns among each video, which reflect the videos’ narrative and semantic natures. The AV video presents results that could be described as following a rational curve trend, with a fast rating increase in the beginning of the presentation followed by a progressive deceleration towards the end of the video. This pattern suggests that the video produces an increase in arousal in the beginning, which is accompanied by a higher frequency of responses compared to the other video modalities, followed by a habituation phase, in which we observe a decrease in the responses. The rating curve of the LS video resembles a line with a constant increase rate; this pattern suggests that it is positive-valenced stimuli, presenting lower arousal than the AV video.

The physiological pattern underlying the duration of the CM video resembles a polynomial curve, beginning with few responses until 15 s, then establishing a descending pattern until nearly 50 s, when inversion occurs, presenting a fast rating increase. This three-phase pattern is common in comedy narratives: the presentation of the characters and the context happens in the beginning and then consecutive events create a minor tension, which is liberated by the comedic “gag” itself (Neale and Krutnik, 1990).

According to the incongruity-resolution theory, humor is based on a two-stage process: incongruity detection, which involves the detection of an incongruous element among two or more compatible events (e.g., a character who is in building on fire, has his body covered with needles and has to jump onto a safety net to save himself); and resolution, in which the incongruent element is linked in a meaningful way to rest of narrative, resolving the incongruity, e.g., the character has to choose between dying or facing a great deal of pain, (Uekermann et al., 2007). In this sense, humor processing involved in comedy videos is highly dependent on the informational aspects of the narrative, which encompasses several elements of social cognition, such as role comprehension, Theory of Mind, understanding of context, understanding of sociocultural norms (Uekermann et al., 2007; Vrticka et al., 2013; Chan et al., 2016) along with affective reactions and executive functions. These points taken together would explain why the time for the first response was up to three times longer than the other two videos, in which affective reactions related to excitement (in the case of the AV video) and aesthetic appreciation (in the case of the LS video) were involved.

Over 50% of the sample for the AV video presentation responded between 5.5 and 6.5 s. This pattern is explained by the high increase in the mean accumulated rating at the beginning of the video, followed by its decrease at the end. The LS video presented the highest mean rating response, with four peaks in the transition between scenes, in which nearly 50% of the sample responded, resembling a sawtooth pattern. Taking this into account, perhaps if the video was comprised of a single scene, this pattern would have been different. Interestingly, for the AV video, the participants tended to make the greatest number of evaluations at the beginning of the video. The CM video presented a percentage of overall response of about 10% less than the other videos, suggesting that comedy involves cognitive and social elements that might be more influenced by individual variability, resulting in less response concomitance, a pattern aligned with the literature (Vrticka et al., 2013; Chan et al., 2016). The results showed that for all videos, there is at least one moment in which more than 40% of the sample responded at the same second, indicating that the time-dependent approach is sensitive and valid to assess group trends over time.

The third and final phase allowed for a detailed investigation of liking at each instant of the video, particularly at moments of maximum and minimum ratings. In keeping with the previous results, the adventure video presented two peaks at the beginning of the video, indicating that these moments were marked with a higher frequency of responses.

The landscape video presented the highest maximum rating. This indicates that a peak of phenomenological well-being derives from calmer and less demanding stimulus, which may be determined by semantic qualities or by the context, as one may argue that in an experimental setting, a relaxing stimulus becomes particularly rewarding. Regarding the comedy video, there were three moments in the middle of the narrative with a greater frequency of responses of negative valence. On the other hand, a higher density of positive responses was observed at the end of the narrative, during the comedic “gag,” corroborating the results found in the other analyses.

### Physiological Measures

#### Electroencephalography

The variable selection for EEG data analysis was made using variables which had some representation in the field, according to our systematic review (submitted). We opted for this approach to deal with overfitting issues and allow direct comparisons with the literature, which was particularly important, given that application of this method to evaluate the psychophysiological correlates of liking videos is innovative.

Univariate analyses presented significant differences in most of the selected variables. The ones with the greatest difference between the EEG power in maximum and minimum ratings were theta in F1 for the adventure, alpha in P4 for the comedy and theta in P4 for the landscape video. These results are aligned with previous findings relating frontal asymmetry and parietal asymmetry, which are consistent with emotional regulation involved in approach-withdraw processing and valence coding (Davidson and Tomarken, 1989; Schellberg et al., 1990; Vecchiato et al., 2010, 2011; Koelstra et al., 2012).

The results for the adventure video are coherent with the literature on affective reactions induced by videos (Schellberg et al., 1990; Vecchiato et al., 2010, 2014; Koelstra et al., 2012; Kortelainen et al., 2015; Güzel Aydin et al., 2016), especially in terms of variables that represent valence and arousal, except for beta in Fp2 (Koelstra et al., 2012), indicating that these variables are associated with liking encoding in this thematic category, at least from the indicators that have already been mapped. In contrast, only six of 17 EEG variables in the comedy video presented significant results that were consistent with the literature in affective reactions induced by videos, more specifically: theta in F1, alpha in P4, alpha and gamma in F2, beta in F2 and P3, being the first four variables mainly involved in valence processing (Schellberg et al., 1990; Vecchiato et al., 2010; Silberstein and Nield, 2008; Vecchiato et al., 2014; Koelstra et al., 2012; Kortelainen et al., 2015) and the final two involved in the processing of both valence and arousal dimensions (Koelstra et al., 2012; Kortelainen et al., 2015). As applied to the AV video, the landscape video presented only three variables that were not aligned with the literature, more specifically: theta in F7, alpha in F8 and beta in C4, with the first variables related to valence (Schellberg et al., 1990; Vecchiato et al., 2010, 2011, 2014) and the last one to arousal processing (Koelstra et al., 2012).

Discrepancies in the literature are expected, given both the wide range of factors that were analyzed and the time-dependent method employed for subjective liking assessment. The ability to collect evaluative responses over time avoids methodological limitations of processes resulting from overall rating at the end of the video, since this tends to dismiss the impact of the intermediary events and give disproportional importance to the beginning and end of the video (Vallar and Papagno, 1986; Baddeley, 2010).

In that vein, the comedy video presented more discrepant results, which, at least to a certain point, may be a consequence of the traditional methodologies that access likeability at the end of the video (Fernández et al., 2012). The adventure and landscape videos presented results that agreed with the literature for approximately 80% of the evaluated variables. This is likely the case because the results did not measure the participant’s interest at a very specific moment (e.g., the gag, as found in the comedy video).

#### Electrocardiography

Univariate analyses showed significant differences in PCR for adventure and comedy, with inverted results for each video; a higher cardiac frequency preceding the maximum rating and lower frequency preceding the minimum rating for the adventure video and the opposite for the comedy video. The increase in cardiac frequency is usually associated with an increase in levels of arousal in response to an stimulus (Fernández et al., 2012), and its association to valence is still controversial; some authors found positive correlations (Gomez et al., 2005; Codispoti et al., 2008; Kortelainen et al., 2015) while others found negative correlations (Vecchiato et al., 2010, 2014; Golland et al., 2014). In this article, the higher likeability scores in adventure was associated with an increase in arousal before the response, which is compatible with the perspective that the more exciting an adventure video, the better it is.

On the other hand, it is possible that in the CM video, the increase in arousal preceding the lower ratings was associated with negative valence feelings, such as anger, tension, and distress. Considering the content of the CM video, it is plausible to argue that such feelings were experienced at different levels by the participants due to their own subjectivities in terms of the context. The video selected for this experiment presented scenes in which one of the characters was receiving acupuncture treatment, with half of his body covered by needles. The story climax occurs when a fire starts breaks out in the building and he must decide whether he will jump through a window onto a safety net held by firefighters, piercing his whole body, or stay in the building. Thus, the increase in arousal could be a response to the increase in expectation and distress evoked by the scene. In this sense, the deceleration of the cardiac frequency might be associated with the relaxation after the climax, elicited by the gag, or even comparison to a more neutral state.

#### Eye Tracking

Univariate analyses showed significant differences in pupil diameter for landscape and comedy. In both cases, this suggests smaller diameters were associated with higher ratings. This finding was also verified by Bradley et al. (2008) in a seminal study of affective reactions to images. Also, it is worth noting that the latency in the CM video is approximately 400 ms lower than in the LS video. Pupil size is also related to cognitive workload (Just et al., 2003), since the cognitive demand of resources involved in judging a comedy must be higher due to the aforementioned aspects, it is reasonable that the latency and pupil diameter to be larger in this video.

### Predicting Time-Dependent Subjective Likeability

The adoption of a multiple regression linear model, a classical technique, relied on its clear interpretability of the weights of each predictor over the dependent variable, directly answering the questions of “what is the best combination” and “what is the corresponding importance of each physiological measure to predict liking for stimuli with distinct semantic categories while using a time-dependent technique.” All models presented significant results and were able to explain the difference between the maximum and minimum rating with 30% more accuracy than by chance (adj. *R*^2^ > 0.3).

In the final adventure model, two variables presented higher importance: beta in C4 and beta in P4, respectively with a time delay of 1367 ms and 1055 ms. The relative weight of beta in C4 was slightly higher than in P4. According to the literature, the increase of beta in the right central and parietal region is related to both arousal processing and valence encoding (Schellberg et al., 1990; Koelstra et al., 2012; Kortelainen et al., 2015). In this sense, according to the correlates obtained using non-time-dependent measures, both variables are representative of valence and arousal, indicating a strong association of these dimensions with the AV video, suggesting that beta in the right centro-parietal area can be a relevant indicator for investigating liking in videos where high arousal and valence are positively correlated.

ECG was excluded from the final model for its redundancy with other variables. Couto et al. (2015) investigated changes in evoked cardiac responses related to affective stimuli and found significant results on the right parietal region. In this sense, it is possible that the electrical activity in this region could be redundant to the ECG activity, which corroborated the “V” shape relationship when considered alone.

Despite the initial comedy model being the only one to include all physiological measures (EEG, ECG, and ET), it was the one that presented a final model which needed the fewest variables to explain the differences in minimum and maximum liking. Indeed, with only the information on the spectral power in alpha in Fp2 at 688 ms before the rating response, it is possible to predict the likeability score with more than 30% accuracy beyond chance. Interestingly, our results are contrary to the literature (Schellberg et al., 1990; Vecchiato et al., 2010, 2011, 2014), indicating that for this type of video, the increase of alpha in the right frontal region is actually inversely correlated to the valence score.

In contrast to the comedy video, the landscape final model was the one with more variables. The relative importance of each variable in ascending order was: beta in P4, alpha in Fp2, alpha in P4, pupil size, beta in C4 and alpha in F8. Beta in C4 and alpha in P4 presented positive coefficients, whereas the other variables presented negative coefficients. Individually, beta in C4 and P4 are associated with valence and arousal processing (Koelstra et al., 2012), a negative correlation between the rating and beta in P4 can represent lower arousal, following the inverse logic of the adventure video. Alpha in P4 was also associated with valence processing (Schellberg et al., 1990; Koelstra et al., 2012; Kortelainen et al., 2015). In contrast to the literature, our findings show an inverse relationship between alpha power increase in the right frontal region and likeability ratings (Schellberg et al., 1990; Vecchiato et al., 2010, 2011, 2014). The landscape final model was the only one to employ measures other than those derived from the EEG, and as on the univariate analysis, the pupil size has shown an inverse relation to the likeability rating.

### A Time-Dependent Method for Assessing the Physiological Correlates of Liking

To our knowledge, only one study evaluating the physiological correlates of affective reactions induced by videos made concomitant subjective evaluations along with the presentation of the stimuli (Golland et al., 2014). However, the method employed by Golland et al. (2014) have two main differences from the one presented in this study: (1) the assessed variable in our study was likeability, whereas their study evaluated emotional arousal; and (2) their equipment registered ratings ranging from 0 and 270°, with lower and upper thresholds, whereas our equipment allowed the participants to give ratings without any predefined limit, allowing analysis using individual evaluative intervals.

The search for objective measures of liking is still not possible without the employment of declarative measures, at least for experimental control. In this sense, the use of evaluation techniques which allow the assessment of variability along time can provide a new ground for affect detection. Our results indicate that the employment of time-dependent measures can provide more information regarding semantic and other time-dependent features of the stimulus, such as its narrative aspects, which cannot be assessed by the overall evaluation approach mainly used in the field. Notwithstanding, the time-dependent approach is much more complex and time-consuming, needing different processing steps to be conducted. However, it may provide insightful information from the dynamic changes of liking along the stimulus presentation. In this sense, researchers should consider whether to use this approach, based on their objectives.

The chosen categories were an adventure, a nature landscape, and comedy, as they present a clear narrative and structure, however, the methodological approach presented in this study could be extended to any video. We believe that this approach can be applied to the evaluation of the physiological correlates of other psychological phenomena, such as discourse reliability, sensorial perception, etc., as long as it can be understood by the subject as one single dimension. Additionally, as the methodology is based on internal differences for the same stimuli, it can be better suited for videos which are not so discrepant in terms of their valence and arousal.

### Study Limitations

Regarding the study design and sample, there are common limitations related to correlation and exploratory studies. The first aspect is the sample size and its uniform nature, which makes generalization of the results difficult. Another limiting aspect regarding the sample is that the analyses did not consider gender differences, due to the sample size and imbalance of groups.

Regarding the stimuli selection, a potential limitation of this study is that only one 1-min video was selected for each category, without referring to a normative database, such as the Emotional Movie Data Base (EMDB; Carvalho et al., 2012) or Database for Emotional Analysis using Physiological Signals (DEAP; Koelstra et al., 2012), since the videos in these databases were collected from music video clips or Hollywood movies, there would be a high probability that the participants had already seen it and therefore biasing their evaluation.

Regarding the physiological measures, each modality has its own strengths and weaknesses. Therefore, by employing them together, both advantages and disadvantages were added together (that is, the limited spatial resolution of the EEG, loss of ET acquisition and muscle artifacts in ECG, just to name a few), which resulted in loss of information, given that only acceptable data was used in the study. Despite using the same monitor settings throughout the entire experiment, it was not possible to correct the brightness in all scenes across stimuli, which may have influenced the pupillometry.

In regard to the likeability assessment, as with any self-reported measure, the results are widely influenced by individual variability and this study was not an exception (in fact, applying time-dependent measurements makes individual variability stack over time). In addition, there was no comparison with any standard evaluation methods for likeability assessment, such as SAM, or even a global scaling technique.

In terms of the predictive model, it is important to note that not all existent indicators found in the literature were investigated [such as skin conductance level (Vecchiato et al., 2010) or body posture (Ramsøy et al., 2017)] but just a few indicators derived from EEG, ECG and ET which had a clear correspondence in the literature. Nevertheless, it was a necessary measure to employ the linear regression. Finally, we employed multiple regression linear models, which assume that there is no measurement error, and therefore its results should be taken with a certain caution.

### Final Comments and Future Directions

With this study, our team attempted to advance one of the most traditional questions of psychology, which is liking, from an updated perspective, in which data is highlighted and the phenomenon is approached in a more careful and methodologically-sound fashion. We proposed the use of a time-dependent approach for mapping the psychophysiological correlation of subjective liking, induced by stimuli with marked semantic differences. This allowed the investigation of narrative nuances for each stimulus, along with many other time-related aspects, providing a noteworthy alternative to the traditional approach used in most studies in the field of affective psychophysiology and affective computing.

Some observations can be determined based on the findings:

1. First, is the need for a methodological refinement phase, enlarging the sample and allowing other groups to collaborate and work on this data, following the steps of DEAP (Koelstra et al., 2012) and EMDB, applying this method on other constructs and stimuli.
2. After the consolidation of the method for the healthy adult population, it is possible to make comparisons with individuals of other cultures, ages, and clinical populations.
3. Another branch would be applying these methods for audiovisual and marketing research, given the potential of this technique to improve advertising and non-advertising videos.
4. Finally, a generalist approach should not be used when assessing audiovisual narrative appreciation from a scientific perspective, given that different thematic categories may demand diverse affective parameters of evaluation. The case of horror movies may be especially interesting, as reactions traditionally associated with displeasure can be argued to be, at least in principle, drivers of positive appreciation for such a category.

## Conclusion

This is the first study to employ time-dependent methods to assess likeability of videos and its psychophysiological correlation in a multimodal (i.e., EEG, ECG and ET) and context- sensitive setup. The use of a time-dependent measure can provide valuable information, such as narrative nuances, sample response over time and scene relevance, which cannot be assessed through traditional methods. Conversely, it is possible to establish the physiological correlates of likeability in a much more precise manner, by pinpointing the most relevant moments of the video and using their corresponding physiological patterns in order to evaluate not only which combination of indicators has the best predictive power, but also the optimal time interval. Despite the methodological limitations of the current study, these findings have important implications for the field of consumer and affective neuroscience, suggesting that there is a considerable difference in the psychophysiological correlates of stimuli with different contextual properties and that the use of time-dependent methods to assess videos should be considered as best practices.

## Ethics Statement

This study was carried out in accordance with the recommendations of the National Health Council of Brazil (CNS 510, from April 07 of 2016), ethics committee for research involving human beings from the Institute of Psychology of the University of Sao Paulo. The protocol was approved by the ethics committee for research involving human beings from the Institute of Psychology of the University of Sao Paulo. All subjects gave written informed consent in accordance with the Declaration of Helsinki.

## Author Contributions

HA: study design, data processing and analysis, manuscript writing. MC: study design, manuscript review. JG and PS: manuscript review. EO: data processing and analysis, manuscript review. AD: study design, manuscript writing, manuscript review.

## Conflict of Interest Statement

The authors declare that the research was conducted in the absence of any commercial or financial relationships that could be construed as a potential conflict of interest.

## Footnotes

^1^The edited versions of the videos can be found at http://bit.ly/video_FHN
