# Supplementary material for: Neural Correlates of Preference: A Transmodal Validation Study
Source: Front Hum Neurosci. 2019 Mar 18;13:73. doi: 10.3389/fnhum.2019.00073 (PMC6431660; doi:10.3389/fnhum.2019.00073)
Supplement: Supplementary file 1 [file Data_Sheet_1.docx]

Supplementary Material

# Physiological measures

## Electroencephalography

**Supplementary Table 1**

The variable which presented the larger temporal latency between the response and the moment of maximum power difference between minimum and maximum likeability ratings were gamma in F8 for AV and LS videos and theta in F7 for CM video. Conversely, the variables which presented the smallest temporal latency for the AV video were alpha in F2, gamma in Fp2 for CM video and beta in Fp2 for LS video. The frequency bands which presented the largest latency were gamma for AV (-1273.33 ms), theta for CM (-1441.33ms) and alpha for LS (-618.33ms); whereas the frequency bands that presented the smallest latency were alpha for AV (-406.5ms), gamma for CM (-492ms) and beta for LS (441.5ms).

Regarding the channels with greater and minimal average latency for each video, respectively, the following results were observed, AV video: C4 (-1363ms) and F2 (-479.33ms); CM video: F7(-1492ms) and F8 (-117ms); LS video: F1(-1035ms) and P3 (-133ms). On average, the AV video presented a greater latency than the other videos (-1086.53ms), followed by the CM video (-745.56ms) and the LS video (-500.8ms). Regarding hemisphere latency, AV (right: -1091.56ms and left: -1086.53ms), CM (right: -532,94ms and left: -1000.7ms) and LS (right: -429.67ms and left: -607.5ms).

The greatest difference the EEG power preceding the minimum and maximum rating response was in theta in F1 for the AV video, in alpha in P4 for CM video and theta in P4 for the LS video; conversely the smallest difference for the AV video was beta on Fp2; for CM video was theta in Fp1 and theta in F7 for the LS video.

The frequency bands which presented the largest average difference were theta in the AV video (17648μV^2^), gamma in the CM video (-886,67μV^2^) and alpha in the LS video (3270.33μV^2^); conversely the frequency bands that presented the smallest difference were gamma for AV (2444.67μV^2^), theta for CM (-28224μV^2^) and theta for LS (-646,33μV^2^). Regarding the channels with greater and minimal average difference for each video, the following results were observed, AV: Fp1 (21200μV^2^) and P3 (982μV^2^); CM: F1(7971μV^2^) and Fp1(-63711μV^2^); LS: F1 F1(13378μV^2^) and F7(-32843μV^2^). On average, the AV video presented a larger difference than the other videos (6334.92μV^2^) followed by the CM video (-13.57μV^2^) and the LS (-8697.6μV^2^). Regarding hemisphere average difference, the following results were observed, AV (right: 3658.36μV^2^ and left: 10349.75μV^2^), CM (right: -3980.72μV^2^ and left: -10349,75 μV^2^) and LS (right: 2083.22μV^2^ and left: -3158.75μV^2^).

## Electrocardiography

**Supplementary Figure 1**

The maximum average cardiac response difference between the preceding moments of maximum and minimum likeability ratings were 3.704±8,476 bpm at -4520 ms (z (1) = 2.163, p = 0.030) in the AV video; -3.845±8.611 bpm at -4074 ms (z (1) = 2.163, p = 0.030) in the CM video and -1.638±8.411 bpm at -0.39 ms (z (1) = 0.501, p = 0.616) in the LS video.

**Supplementary Figure 2**

## Eye tracking

The maximum average pupil size difference between the preceding moments of maximum and minimum likeability ratings were -0.057 ±0.422 mm at -883 ms (z (1) = 0.820, p = 0.412) in the AV video; -0.192±0.408 mm at 1801 ms (z (1) = 2.163, p = 0.030) in the CM video and -0.122±0.620 mm at -1.383 ms (z (1) = 2.254, p = 0.024) in LS video.

**Supplementary Table 1:** EEG power maximum differences and corresponding time between the 1.5 seconds preceding the maximum and minimum ratings for each video.

|  | **Adventure** | | | | **Comedy** | | | | **Landscape** | | | |
| --- | --- | --- | --- | --- | --- | --- | --- | --- | --- | --- | --- | --- |
| **Variable** | **Z(1)** | **p-value** | **time (ms)** | **Mean difference** | **Z(1)** | **p-value** | **time(ms)** | **Mean difference** | **Z(1)** | **p-value** | **time(ms)** | **Mean difference** |
| Fp1 θ | 1.981 | 0.048 | -660 | 21.2±59.15 | -2.049 | 0.040 | -1367 | -63711±180075 | - | p>0.05 | - | - |
| F7 θ | - | p>0.05 | - | - | -2.004 | 0.045 | -1492 | -28932±60975 | -2.004 | 0.045 | -496 | -32843±103595 |
| F1 θ | 1.981 | 0.048 | -1242 | 14096±39829 | 1.981 | 0.048 | -1465 | 7971±43004 | 2.118 | 0.034 | -1035 | 13378±37204 |
| P4 α | - | p>0.05 | - | - | 1.981 | 0.048 | -297 | 19607±43633 | 2.095 | 0.036 | -1113 | 13089±36616 |
| P4 θ | - | p>0.05 | - | - | - | p>0.05 | - | - | 2.049 | 0.040 | -234 | 17526±53010 |
| O1 θ | - | p>0.05 | - | - | - | p>0.05 | - | - | - | p>0.05 | - | - |
| O2 θ | 2.118 | 0.034 | -1363 | 6500±18163 | -2.983 | 0.003 | -188 | -13070±31016 | 1.981 | 0.048 | -395 | 7231±22009 |
| Fp2 α | 2.095 | 0.036 | -688 | 6145±16867 | -2.664 | 0.008 | -688 | -10367±25580 | 2.049 | 0.040 | -254 | 16546±43618 |
| F8 α | - | p>0.05 | - | - | -2.118 | 0.034 | -762 | -50134±236200 | -2.095 | 0.036 | -488 | -19824±50400 |
| F2 α | 2.049 | 0.040 | -125 | 5653±18693 | 2.300 | 0.022 | -1109 | 10404±24671 | - | p>0.05 | - | - |
| Fp2 γ | 2.573 | 0.010 | -1371 | 2449±4664 | -2.118 | 0.034 | -113 | -1306±4056 | 2.414 | 0.016 | -141 | 1152±3407 |
| F8 γ | 2.733 | 0.006 | -1406 | 3194±5821 | -2.300 | 0.022 | -117 | -2376±6611 | 2.459 | 0.014 | -1281 | 5310±14692 |
| F2 γ | 2.232 | 0.026 | -1043 | 1691±6000 | 2.323 | 0.020 | -1246 | 1022±3235 | 2.368 | 0.018 | -254 | 552±1265 |
| Fp2 β | -2.004 | 0.045 | -965 | -1464±11241 | -2.118 | 0.034 | -211 | -3476±14697 | 1.981 | 0.048 | -105 | 3772±9890 |
| F8 β | 2.414 | 0.016 | -1148 | 4097±9300 | - | p>0.05 | - | - | 2.141 | 0.032 | -535 | 2828±15599 |
| F2 β | 2.095 | 0.036 | -270 | 1776±7023 | 1.981 | 0.048 | -1348 | 3581±7615 | 2.095 | 0.036 | -258 | 1982±7541 |
| C4 β | 2.004 | 0.045 | -1367 | 3943±9316 | -2.801 | 0.005 | -637 | -4567±9073 | -2.641 | 0.008 | -145 | -2590±5966 |
| P3 β | 2.118 | 0.034 | -1148 | 982±6491 | 2.414 | 0.016 | -445 | 5201±12208 | 2.186 | 0.029 | -133 | 1633±4329 |
| P4 β | 1.981 | 0.048 | -1055 | 2445±8058 | -2.232 | 0.026 | -684 | -3824±7978 | 2.163 | 0.031 | -1195 | 3330±7669 |
| O1 β | 2.368 | 0.018 | -1266 | 5121±12865 | -2.323 | 0.020 | -172 | -10183±21634 | 1.981 | 0.048 | -766 | 5197±17861 |


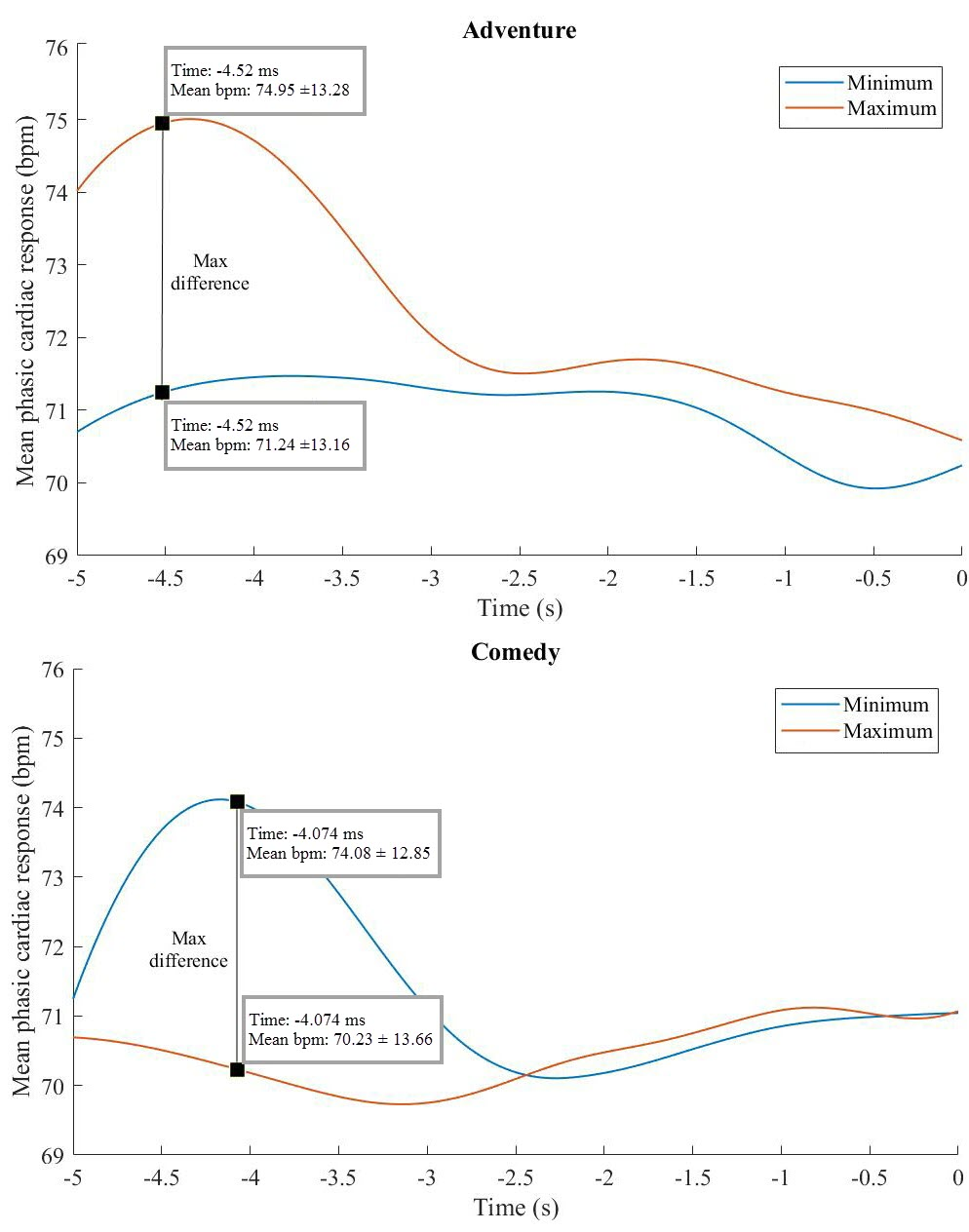


**Supplementary Figure 1:** Comparison of the mean phasic cardiac response 5s preceding the moments of maximum and minimum rating for the adventure and comedy videos.


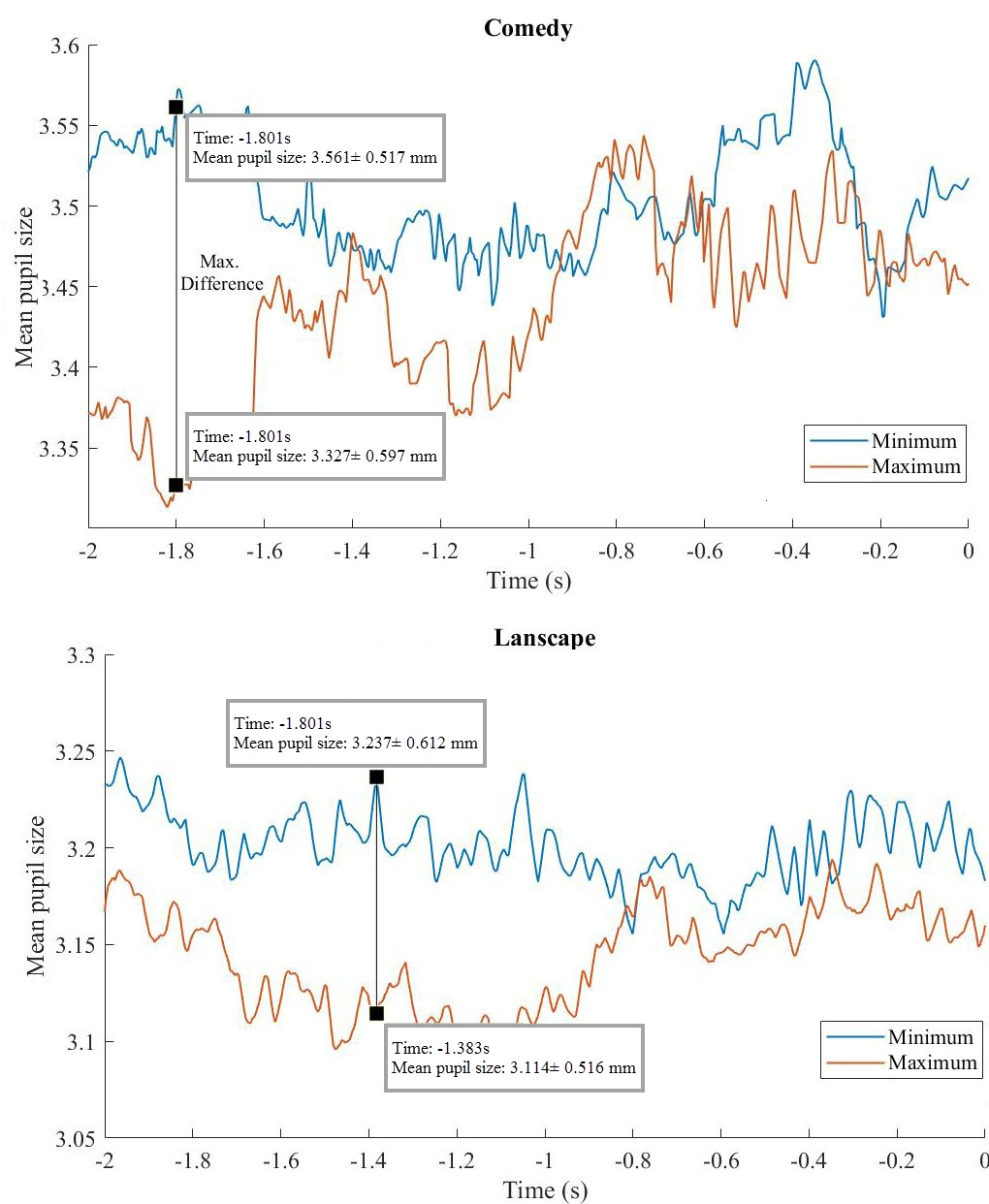


**Supplementary Figure 2:** Comparison of the mean pupil size 2s preceding the moments of maximum and minimum rating, for the comedy and landscape videos.
